# Characterization of Mucosal Disaccharidases from Human Intestine

**DOI:** 10.3390/nu9101106

**Published:** 2017-10-10

**Authors:** Mahdi Amiri, Hassan Y. Naim

**Affiliations:** Department of Physiological Chemistry, University of Veterinary Medicine Hannover, D-30559 Hannover, Germany; mahdi.amiri@tiho-hannover.de

**Keywords:** intestinal disaccharidases, enzyme activity, pH profile, thermal activity profile

## Abstract

In this study, we used a brush border membrane (BBM) preparation from human small intestine to analyze the proportion and the activity of major intestinal disaccharidases, including sucrase-isomaltase (SI), maltase-glucoamylase (MGAM) and lactase-phlorizin hydrolase (LPH). SI, MGAM and LPH respectively constituted 8.2%, 2.7% and 1.4% of total BBM protein. The activity of SI and LPH decreased threefold after purification from the brush border membrane, which highlights the effect of membrane microdomains on the functional capacity of these enzymes. All of the disaccharidases showed optimal activity at pH 6, over 50% residual activity between pH 5 to pH 7, and increasing activity with rising temperatures up to 45 °C, along with a stable functional structure. Therefore the enzymes can withstand mild intraluminal pH alterations with adequate function, and are able to increase their activity with elevated core body temperature. Our data provide a functional measure for characterization of intestinal disaccharidases under different physiological and pathological conditions.

## 1. Introduction

The final step of carbohydrate digestion is performed on the intestinal mucosa by the action of intestinal disaccharidases. Sucrase-isomaltase (SI), maltase glucoamylase (MGAM) and lactase-phlorizin hydrolase (LPH) are the major disaccharidases of the small intestine that hydrolyze di- or oligosaccharides to monosaccharides for absorption [1]. SI with α-1,2, α-1,4 and α-1,6 activities can hydrolyze sucrose, maltose, isomaltose and maltooligosaccharides, which are either ingested or produced by the endo-α-amylase activity of salivary and pancreatic amylases on starch in the gut [2]. MGAM has α-1,4-glucosidase activity and contributes to digestion of maltose and maltooligosaccharides [2]. LPH with β-galactosidase activity is the only enzyme to hydrolyze milk sugar—lactose—in the intestinal lumen [1,3].

These multifunctional disaccharidases are membrane-anchored and heavily *N*- and *O*-glycosylated proteins, and are synthesized as single polypeptides at the ER membrane, trafficked along the secretory pathway and sorted onto the apical surface of the epithelial cells with high fidelity [4,5,6]. *O*-glycosylation is a crucial event, dictating the polarized sorting of SI to the apical membrane [7,8]. SI and MGAM are type II membrane glycoproteins with two active subunits at their luminal part. The two enzymes share a high degree of homology (58.4% identity and 74.3% similarity, EMBOSS Needle), and are thought to originate from a common ancestral gene [9]. Targeted sorting of SI to the apical surface of the intestinal epithelial cells involves *O*-glycosylation of its stalk region and recruitment of the mature form of SI in cholesterol-sphingolipid enriched lipid rafts at the *trans*-Golgi network (TGN) [7,8]. At the luminal surface, SI is cleaved by trypsin in between its constituent subunits, which remain further associated via non-covalent interactions [4]. By virtue of the striking structural homologies between SI and MGAM and the similar biosynthetic pathways [5] it can be assumed that MGAM is also trafficked to the apical membrane via similar sorting mechanisms.

LPH is a type I membrane glycoprotein that is produced as a pro-peptide with four homologous domains. The non-functional domains I and II at the N-terminus are removed by two sequential proteolytic activities at the TGN and the cell surface [10]. The functional LPH is composed of domain III with phlorizin hydrolase activity, domain IV with lactase activity, a transmembrane region and a small cytoplasmic tail at the C-terminus [10]. Unlike SI, LPH does not interact with lipid rafts on its way to the apical membrane, and is packaged in vesicles at the TGN with a different membrane composition from those that transport SI [11].

Deficiencies in the function of disaccharidases either as a result of genetic mutations or as a consequence of organ pathologies are associated with maldigestion of carbohydrate and gastrointestinal intolerance symptoms such as diarrhea, flatulence and abdominal pain [12].

In the current study, we have characterized the luminal expression and the activity levels of intestinal disaccharidases. Additionally, the effects of pH or incubation temperature on disaccharidase activities have been analyzed. Our data provide a more comprehensive insight into the amount, activity and functional structure of intestinal disaccharidases, and can be used to explain the influence of pathological intraluminal conditions on the carbohydrate digestion capacity of the small intestine.

## 2. Materials and Methods

HBB 1/909/34/74 anti-LPH and HBB 3/705/60 anti-SI antibodies [13], as well as the human brush border membrane (BBM) preparation, were kindly provided by Dr. Hans-Peter Hauri and Dr. Erwin E. Sterchi (Bern, Switzerland). The BBM preparations were isolated from intestine of a healthy human kidney donor subject according to Schmitz et al. [14], and were approved by the ethical committee at the University of Bern. Dr. Buford L Nichols (Baylor College of Medicine) generously provided HSI2 antibody against human SI [15] and LAMA 1/207/140/12, LAMA 1/77/6/2/1 and LAMA 1/127 antibodies against human MGAM. MLac1, MLac6 and MLac10 antibodies against LPH [16] were kindly provided by Dr. Dallas Swallow (Medical Research Council, London). 

The BBM sample was solubilized in 25 mM Tris buffer pH 8 containing 50 mM NaCl, 0.5% Triton X-100 and 0.5% sodium deoxycholate supplemented with a mixture of protease inhibitors. Then, SI, MGAM and LPH were separately immunoprecipitated from the lysate. A part of the immunoprecipitated proteins was analyzed by SDS-PAGE, followed by either silver nitrate staining or immunoblotting to determine the BBM constituent of the target disaccharidase. The other part of the immunoprecipitants was used for enzyme activity measurement of the purified protein. Specific activity for each disaccharidase was calculated in units per milligram of pure protein. Additionally, sucrase, isomaltase, maltase and lactase activities were measured in unsolubilized BBM samples and the specific activities for these activities were determined based on the BBM content of the corresponding enzyme(s). The detailed experimental approach is provided in Appendix B. Data analysis was performed with Microsoft Excel.

## 3. Results

### 3.1. Content and Activity of Intestinal Disaccharidases

Human intestinal brush border membrane preparation was solubilized, and SI, MGAM or LPH were immunoprecipitated using monoclonal antibodies. The immunoprecipitants were used to determine the amount of intestinal disaccharidases, as well as their specific activity for the corresponding substrates (Table 1), as described in the methods (Appendix B). The data showed that SI, MGAM and LPH constitute 8.2%, 2.7% and 1.4% of total proteins in the BBM sample, respectively. Allocating almost 11% of total BBM protein to α-glucosidases (SI and MGAM) is noticeable, and is concomitant with the high proportion of carbohydrates in the daily diet. We further tested the unsolubilized BBM sample for sucrase, isomaltase, maltase and lactase activities, and calculated the specific activities based on the amounts of the corresponding enzymes as determined earlier. For BBM maltase activity, which is shared between SI and MGAM proteins, the collective amount of these proteins, including their abundance, was considered (Table 1). In comparison to the activity levels recovered from unsolubilized BBM, SI and LPH showed an almost threefold decrease in activity after solubilization and immunoprecipitation. Sodium deoxycholate, which was included in addition to Triton X-100 in the lysis buffer, efficiently disrupts membrane microdomains, and dissociates protein interactions for a more specific isolation of the target proteins. These data highlight the crucial effect of the membrane milieu, particularly the membrane microdomains, on the functional structure of intestinal disaccharidases. The complex *N*- and *O*-glycosylated SI is associated with cholesterol-sphingolipid enriched membrane microdomains at the *trans*-Golgi network, and sorted to the apical surface of the intestinal epithelial cells with high fidelity [11]. Extraction of SI from these membrane microdomains is shown to substantially reduce its functional capacity [17]. Although mature LPH associates with a different type of membrane microdomains than those with SI, our data show that the ultimate outcome of such associations in enhancing the functional capacity for both of the disaccharidases is the same.

### 3.2. The Effect of pH and Temperature on the Function of Intestinal Disaccharidases

Temperature, pH, and type and strength of ions in the enzyme milieu are potential factors that can influence the function of an enzyme [18]. In the intestinal lumen of healthy human subjects, the pH ranges from 6.15 to 7.88 with a gradual increase along the proximal-distal intestinal axis [19]. Moderate changes in pH can influence the ionization of the amino acid residues included in the catalysis or, occasionally, substrate binding, and can therefore modulate enzyme activity. Extreme deviations from physiological pH can further influence the native structure of the enzymes, and can consequently affect their interactions or functions. Disorders such as small bowel syndrome and different forms of enteritis, which chronically alter the intraluminal acid-base homeostasis, can potentially affect the functional efficiency of the intestinal disaccharidases [20]. We have determined the pH profile for different disaccharidase activities from human BBM preparation. For this purpose, enzyme assays with corresponding substrates were performed in phosphate citrate buffer with different pH values ranging from 3 to 8. Interestingly, pH 6 was found to be the common optimum pH for sucrase, maltase and lactase activities from the intestinal BBM. In general, all tested disaccharidases showed over 50% of their optimal-pH-activity between pH 5 to pH 7, which is consistent with the overall intestinal pH [19]. Higher abundance of the disaccharidases in the proximal gut where the pH is closer to their optimum pH provides efficient conditions for carbohydrate digestion in this region. Due to the broad peak of the pH profiles for intestinal disaccharidases, slight deviations from the normal intraluminal pH are not expected to cause substantial alterations in the functional efficiency of intestinal disaccharidases.

**Figure 1:**
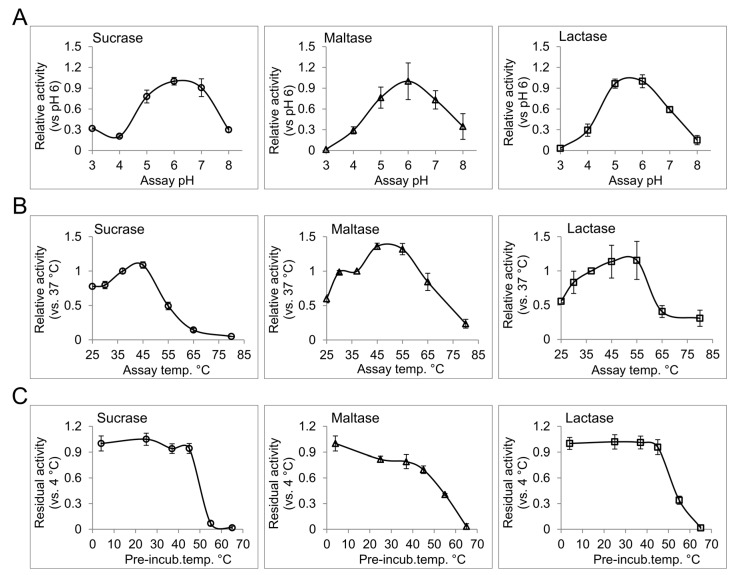
The effect of pH and temperature on the disaccharidase activities from human intestinal brush border membrane preparation. (**A**) The pH profile for sucrase, maltase and lactase activities was determined by measuring the enzyme activities in phosphate citrate buffer with different pH values ranging from 3 to 8; (**B**) The enzyme reactions were performed at different temperatures from 25 °C to 80 °C in phosphate citrate buffer at pH 6 to determine the thermal activity profile; (**C**) The enzyme samples in phosphate citrate buffer pH 6 were treated at the indicated temperature for 1 h and then used to measure the residual activity at 37 °C. Error bars: standard deviation.

The baseline of intestinal temperature has been measured at about 37 °C [21], which can rise to about 40 °C with exercise [22]. To characterize the effect of temperature on the activity and the structural stability of the intestinal disaccharidases, we determined the thermal activity and thermal stability profiles of these enzymes. For the thermal activity profiles of sucrase, maltase and lactase, the activities of the BBM preparations at different temperatures ranging from 25 °C to 80 °C were analyzed (Figure 1B). All of the tested activities showed a gradual increase up to 45 °C or, in the case of lactase, up to 55 °C. A sharp and significant increase for maltase activity (36%, *p* > 0.05 paired *t*-test) from 37 °C to 45 °C was observed, which might be a physiological response to enhance maltose and starch digestion during physical exercise, where intestinal temperature is slightly elevated. Thermal stability profiles for the upper activities are presented in Figure 1C. For this assay, the enzymes were pre-incubated at different temperatures from 4 °C to 65 °C for 1 h, then immediately used for enzyme activity analysis at 37 °C with the corresponding substrates. Taking the 4 °C pre-incubation as reference, sucrase and lactase activities show an outstanding stability up to 45 °C, while maltase activity showed a gradual loss of activity over increasing temperatures, showing about 70% of the reference activity at 45 °C. Here, substantial or almost complete loss of activity at temperatures equal to or greater than 55 °C are in line with the results of thermal activity assay and delineates loss of functional structure for the disaccharidases at these temperatures. Since the BBM preparations comprise the original membrane composition of the intestinal epithelium our results can represent the in vivo conditions with a reliable fidelity. The non-physiological temperatures included in the study have provided more details on the functional structures of the analyzed disaccharidases, and can be used as a measure for comparing their structural-functional features with similar proteins.

## 4. Conclusions

Many gastrointestinal disorders are directly or indirectly associated with the malfunction of intestinal disaccharidases. In this study, we aimed to provide a measure for the amount and activity of the disaccharidases from intestine of a healthy human subject, and characterize the effects of environmental factors, such as pH and temperature, on their function. Our results showed that SI and MGAM in combination comprise about 11% of total BBM proteins, which indicates that a high proportion of mucosal proteins are dedicated to carbohydrate digestion. In previous studies, the activities of intestinal disaccharidases have been normalized to the wet weight of the intestinal biopsies or the total protein content of the sample [23,24], which results in a wide range of normal disaccharidase activities based on expression levels in different individuals [25]. Here, the activities are determined based on the mean disaccharidase content, and provides a more confined reference unit, which is independent of the expression level. This method can be used as a tool for specifying the rationale of disaccharidase insufficiency towards either loss of function [26] or lowered intestinal expression [25] of the enzymes. We identified that extraction of the disaccharidases from the membrane affects their functional capacity; this point should be considered when comparing activity values from different studies. SI, MGAM and LPH have their optimum activity at pH 6, and preserve more than 50% of their activity up to pH 7, which is consistent with the intraluminal pH in the small intestine. Accordingly, slight pH variations in the intestinal lumen caused by chronic disorders are not expected to substantially affect the function of the intestinal disaccharidases per se. The activity of the intestinal disaccharidases increased with an increase in temperature up to 45 °C, with an acceptable stability. This effect could enhance the functional efficiency of these disaccharidases when physical exercise increases the core body temperature. The novel data provided in this study expand the current knowledge on the function of intestinal disaccharidases, and could provide a scale for functional characterization of these enzymes under pathological conditions.

## Figures and Tables

**Table 1:** The amount and activity of major disaccharidases from human intestinal brush border membrane preparation.

		SI			MGAM	LPH
Content (% total BBM protein)		8.2 ± 0.7			2.7 ± 1.4	1.4 ± 0.5
Substrate		Sucrose	Isomaltose	Maltose	Maltose	Lactose
Specific activity (U·mg^−1^)	immunopr.	9.5 ± 1.9	5.2 ± 1.4	10.3 ± 3.3	28.1 ± 12.4	1.8 ± 0.3
	BBM	27.8 ± 0.5	16.5 ± 0.8	20.2 ± 0.4		5.6 ± 0.3

The amounts of sucrase isomaltase (SI), maltase glucoamylase (MGAM) and lactase-phlorizin hydrolase (LPH) disaccharidases in the human intestinal brush border membrane (BBM) preparation was determined by SDS-PAGE analysis and reported as percent of total BBM proteins. The activities of these disaccharidases in the BBM sample (natural milieu) or in the immunoprecipitated form were determined using their respective substrate(s).

## Appendix A. Materials

HBB 1/909/34/74 anti-LPH and HBB 3/705/60 anti-SI antibodies [13], as well as the human brush border membrane (BBM) preparation, were kindly provided by Dr. Hans-Peter. Hauri and Dr. Erwin E. Sterchi (Bern, Switzerland). The BBM preparations were isolated from intestine of a healthy human kidney donor subject according to Schmitz et al. [14], and approved by the ethical committee at the University of Bern. Dr. Buford L Nichols (Baylor College of Medicine) generously provided HSI2 antibody against human SI [15] and LAMA 1/207/140/12, and LAMA 1/77/6/2/1 and LAMA 1/127 antibodies against human MGAM. MLac1, MLac6 and MLac10 antibodies against LPH [16] were kindly provided by Dr. Dallas Swallow (Medical Research Council, London, UK).

Horseradish peroxidase-conjugated secondary antibodies, molecular weight standards for SDS-PAGE, and SuperSignalTM West Fermento maximum sensitivity western blot chemiluminescence substrate were obtained from Thermo Fisher Scientific. Protein A-sepharose, protease inhibitors, α-lactose and Triton X-100 were from Sigma-Aldrich (Munich, Germany). Acrylamide, Tris, TEMED, SDS, Dithiothreitol, polyvinyl difluoride (PVDF) membrane, sucrose, and maltose were obtained from Carl Roth GmbH (Karlsruhe, Germany). Isomaltose was purchased from Cayman Chemical (Biomol GmbH, Hamburg, Germany). Glucose oxidase-peroxidase mono-reagent was obtained from Axiom GmbH (Bürstadt, Germany). All other chemicals were of analytical grade.

## Appendix B. Experimental Approach

### Appendix B.1. Determination of BBM Content and Activity of the Disaccharidases

The BBM sample was solubilized in 25 mM Tris buffer with pH 8 containing 50 mM NaCl, 0.5% Triton X-100 and 0.5% sodium deoxycholate, supplemented with a mixture of protease inhibitors. The lysis was completed with gentle mixing for 1 h at 4 °C, and the debris were removed by cold centrifugation at 10,000× *g* for 5 min. Protein A-sepharose beads pre-conjugated with HSI2 (anti-SI), a combination of LAMA 1/207/140/12, LAMA 1/77/6/2/1 and LAMA 1/127 (anti MGAM), or a combination of MLac1 and HBB 1/909/34/74 (anti-LPH), were used to separately immunoprecipitate (IP) the target disaccharidases from the lysate. The immunoprecipitants were washed three times with the lysis buffer and divided into three fractions for enzyme activity measurement (1/10), immunoblotting (1/10) or SDS-PAGE and silver nitrate staining (the rest) analysis.

The IP-beads destined for enzyme activity were further washed and equilibrated in phosphate citrate buffer pH 6, and resuspended in 20 µL final volume of the same buffer. Sucrose (150 mM), maltose (20 mM), isomaltose (60 mM) and lactose (300 mM) substrates were also prepared in phosphate citrate buffer pH 6. For each assay, 20 µL of the substrate was mixed with the immunoprecipitated disaccharidase and incubated at 37 °C for 1 h. The reaction was then terminated by adding 10 µL of 2 mM *N*-butyldeoxynojirimycin, which substantially inhibits intestinal SI, MGAM and, to a lesser extent, LPH [27]. The amount of produced glucose was determined based on the Trinder glucose activity test [28] using glucose oxidase-peroxidase mono reagent from Axiom. The contents of this reagent can also completely inactivate the LPH. For sucrase and lactase, 1 µmol of glucose, and for maltase and isomaltase 2 µmol of glucose liberated in one minute were determined to be one unit of activity.

The BBM content of each disaccharidase was determined with a combination of two SDS-PAGE analyses. In the first analysis, a proportion of the total IP was resolved on 6% SDS-PAGE in parallel with samples of bovine serum albumin (BSA) with different concentrations. The gel was then fixed and stained based on the silver nitrate method [29]. The bands corresponding to disaccharidases and the BSA controls were quantified, and the absolute amount of the target disaccharidase was quantified based on the band intensities. In the second analysis, a sample consisting of one tenth of total immunoprecipitated protein and a BBM sample containing 0.6 µg total protein were used for Western blotting. After transfer, the membranes were blocked in PBS solution containing 0.1% Tween 20 and 5% skimmed milk powder. Then, immunoblotting was performed to detect either SI (HBB 3/705/60 antibody), MGAM (combination of LAMA 1/207/140/12, LAMA 1/77/6/2/1 and LAMA 1/127 antibodies), or LPH (combination of MLac6 and MLac10 antibodies) proteins. The protein bands were visualized by addition of chemiluminescence substrate, and documented using ChemiDoc XRS System (Bio-Rad). The band intensities were quantified using the Quantity One 1-D analysis software (Bio-Rad). The amount of immunoprecipitated disaccharidase, which was determined in comparison to the BSA standard samples, was used as a reference to calculate the band intensity-protein amount ratio for each disaccharidase in different immunoblots. Based on this ratio, the amount of the target disaccharidase in 0.6 µg total BBM sample in the same immunoblot was calculated and reported as a percentage of total existing proteins in the BBM sample.

In parallel to immunoprecipitation, sucrase, maltase, isomaltase and lactase activities were determined from un-solubilized BBM samples with a similar method to that described earlier for the enzyme assay from IP-beads.

The amount of disaccharidase in each enzyme measurement was used to calculate the specific activity in units per milligram. Maltase activity in un-solubilized BBM samples is performed by both SI and MGAM proteins. Therefore in this sample, the respective quantity of both of these disaccharidases in the enzyme sample was used to calculate the specific activity.

### Appendix B.2. The Effects of pH and Temperature on the Activity of Intestinal Disaccharidases

In general, 20 µL of BBM samples containing 0.6 µg total protein for the sucrase and lactase assays, or 0.15 µg total protein for the maltase assay, were mixed with equal volumes of substrate, including 150 mM sucrose, 20 mM maltose or 300 mM lactose, and incubated for 1 h. Then, the reaction was terminated, the amount of the liberated glucose was measured, and the units of activity were calculated as described earlier.

To determine the effect of pH on the activity profile of the disaccharidases, the enzyme and the substrate samples were prepared in phosphate citrate buffers with different pH values, including 3, 4, 5, 6, 7 and 8. The activities were normalized to the average activity at pH 6 for all repeats.

For thermal activity and thermal stability profiles, the enzyme samples and the substrates were prepared in phosphate citrate buffer pH 6. For thermal activity profiles, the reaction was carried out at a range of temperatures, including 25, 30, 37, 45, 55, 65 and 80 °C. The activities were normalized to the average activity of the 37 °C assay for all repeats.

For the thermal stability profile, the enzymes were pre-incubated at different temperatures, including 4, 25, 37, 45, 55 and 65 °C for one hour, then cooled down on ice, mixed with the substrate, and incubated at 37 °C for 1 h to determine the residual activity. The residual activities were normalized to the average activity of the samples pre-incubated at 4 °C for all repeats.
